# Comparing the clinical efficacy of abiraterone acetate, enzalutamide, and orteronel in patients with metastatic castration-resistant prostate cancer by performing a network meta-analysis of eight randomized controlled trials

**DOI:** 10.18632/oncotarget.17741

**Published:** 2017-05-10

**Authors:** Minyong Kang, Chang Wook Jeong, Cheol Kwak, Ja Hyeon Ku, Hyeon Hoe Kim

**Affiliations:** ^1^ Department of Urology, Samsung Medical Center, Sungkyunkwan University School of Medicine, Seoul, Republic of Korea; ^2^ Department of Urology, Seoul National University Hospital, Seoul, Republic of Korea

**Keywords:** castration-resistant prostate cancer, androgen receptor signaling, targeted drug, systematic review, network meta-analysis

## Abstract

Various novel androgen receptor (AR) targeting drugs have been developed recently and have shown beneficial effects on survival in patients with metastatic castration-resistant prostate cancer (mCRPC). However, no consensus has been reached regarding which of these agents provides the most favorable oncological outcomes. Here, we aimed to compare the efficacy of novel AR-targeted agents by performing a network meta-analysis of randomized controlled trials (RCTs). We included eight RCTs for men with mCRPC treated with one of the AR targeting agents: abiraterone acetate, enzalutamide, or orteronel. The primary endpoint was overall survival (OS), while the secondary endpoints were progression-free survival (PFS), prostate-specific antigen (PSA) responsiveness, time to PSA progression, time to first skeletal-related events (SRE), and adverse events (AEs). Pairwise meta-analysis and network meta-analysis were conducted to obtain direct and indirect evidence, respectively. Notably, enzalutamide and abiraterone were significantly associated with improved OS compared with control arms. Enzalutamide was ranked as the most efficacious agent for improving OS (hazard ratio [HR] = 0.71), and abiraterone appeared to be the second-most efficacious drug for this purpose (HR = 0.78). Enzalutamide improved PFS in comparison with control groups (HR = 0.36), but abiraterone and orteronel were not significantly associated with PFS improvements. Enzalutamide (HR = 0.20) and abiraterone (HR = 0.56) were significantly associated with prolonged times to PSA progression as compared with control groups. However, only orteronel was associated with an increased risk of AEs as compared with control groups. In summary, our study can help to guide treatment selection, especially because AR-targeted agents have not been compared directly in head-to-head trials.

## INTRODUCTION

Prostate cancer is the most commonly diagnosed cancer among men worldwide, and is the second most common cause of cancer-specific death in Western men [1]. Although patients with localized prostate cancer are managed using radical surgery or radiation therapy, those with advanced or metastatic prostate cancer can initially be treated with androgen deprivation therapy (ADT) [2]. These patients with advanced cases are usually responsive to ADT, but their disease inevitably progresses to castration-resistant prostate cancer (CRPC), which is defined as disease that is progressive, despite ADT-induced castrate levels of serum testosterone [3].

For patients with metastatic CRPC (mCRPC), taxane-based chemotherapy has been the treatment of choice for over a decade, since the success of the TAX327 trial [4]. However, almost all patients with mCRPC acquire drug resistance and ultimately die within two or three years after treatment with systemic chemotherapy [5]. Until recently, therapeutic options for docetaxel resistance were very limited in patients with mCRPC, but improvements in the understanding of CRPC biology led to the development of novel therapeutic agents that target the androgen receptor (AR) signaling pathway, such as abiraterone acetate and enzalutamide [6]. Because men with mCRPC persistently maintain AR activity, despite castration levels of serum testosterone, novel AR-targeted agents can be effective for men with disease that is already resistant to ADT [7].

Several key phase III randomized controlled trials (RCTs) have revealed that these novel drugs significantly improved the survival of mCRPC patients in either pre- or post-chemotherapeutic settings [8–12]. However, various AR-targeting drugs, including abiraterone acetate, enzalutamide, and orteronel (TAK-700), have shown inconsistent therapeutic effects on oncological outcomes in patients with mCRPC [13]. Thus, no consensus has been reached regarding the agent that provides the best oncological outcomes. Here, we sought to compare the efficacy of the novel AR-targeted agents by performing a network meta-analysis of RCTs. Our network meta-analysis may provide evidence that is crucial to treatment selection for patients with mCRPC, especially because novel AR-targeting agents have not yet been compared with each other in head-to-head clinical trials.

## RESULTS

The eight included RCTs had sample sizes ranging from 183 to 875 patients, with a mean of 541 patients. In total, 4,911 and 3,755 men with mCRPC were randomly assigned to treatment and control arms of the RCTs, respectively. Table 1 summarize the study characteristics of these eight RCTs for our network meta-analysis.

**Table 1:** Study characteristics of eight randomized controlled trials for a network meta-analysis

Author [Reference]	Study name	Year	Journal	Treatment arm (N)	Control arm (N)	Treatment setting	Primary endpoint	Median OS (mon)	Median F/U duration (mon)
de Bono [8]	COU-AA-301	2011	NEJM	Abiraterone plus PD (797)	PD (398)	Post-chemotherapy	OS	15.8	20.2
Ryan [10]	COU-AA-302	2013	NEJM	Abiraterone plus PD (546)	PD (542)	Pre-chemotherapy	OS, radiographic PFS	34.7	49.2
Scher [9]	AFFIRM	2012	NEJM	Enzalutamide (800)	Placebo (399)	Post-chemotherapy	OS	18.4	14.4
Beer [11]	PREVAIL	2014	NEJM	Enzalutamide (872)	Placebo (875)	Pre-chemotherapy	OS, radiographic PFS	35.3	31
Saad [12]	ELM-PC 4	2015	Lancet Oncology	Orteronel plus PD (781)	PD (789)	Pre-chemotherapy	OS, radiographic PFS	31.4	20.7
Fizazi [15]	ELM-PC 5	2015	JCO	Orteronel plus PD (734)	PD (365)	Post-chemotherapy	OS	17	10.7
Shore [20]	TERRAIN	2016	Lancet Oncology	Enzalutamide (183)	Bicalutamide (189)	Pre-chemotherapy	PFS	Not reported	20
Penson [21]	STRIVE	2016	JCO	Enzalutamide (198)	Bicalutamide (198)	Pre-chemotherapy	PFS	Not reported	Not reported

**Figure 1:**
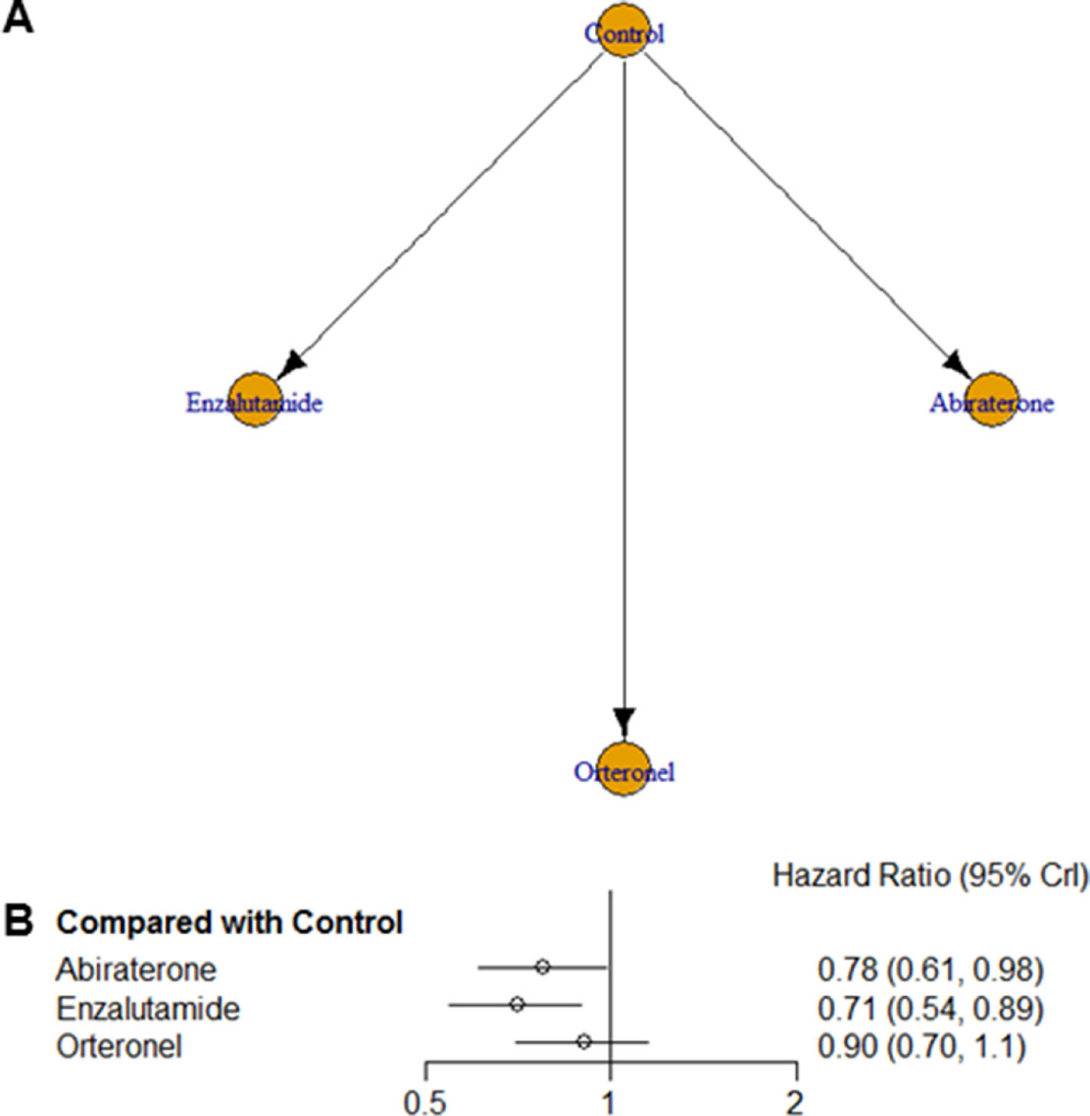
(**A**) Network geometry of the eight randomized controlled trials of novel drugs targeting the androgen receptor signaling pathway. Overall survival was analyzed for patients with metastatic castration-resistant prostate cancer. Arrows indicate studies that were direct comparisons between the agents shown using yellow circles. (**B**) Pooled hazard ratios and 95% credible intervals for overall survival (the primary endpoint of our study).

We performed a network meta-analysis to compare the efficacies of the three AR-targeted agents in terms of overall survival (OS) rates (the primary endpoint of our study). Figure 1A presents the network of potential comparisons for the three different treatments. We were particularly careful when interpreting results for nodes that were poorly connected in this network. Of note, enzalutamide and abiraterone were significantly associated with improved OS in comparison with controls, as shown in Figure 1B. More importantly, enzalutamide was ranked as the most efficacious agent for improving OS rates (Hazard ratio (HR) = 0.71, 95% credible intervals (CrI) = 0.54–0.89), while abiraterone appeared to be the second most efficacious drug for this purpose (HR = 0.78, 95% CrI = 0.61–0.98). Although OS also tended to be better in orteronel groups than in control groups, the difference was not statistically significant (HR = 0.90, 95% CrI = 0.70– 1.10). The details of results for the rankings of these novel AR targeting drugs are summarized in Supplementary Table 1. No inconsistency was observed between the direct and indirect evidence obtained by comparing the results of pairwise and network meta-analyses (Supplementary Figure 1).

**Figure 2:**
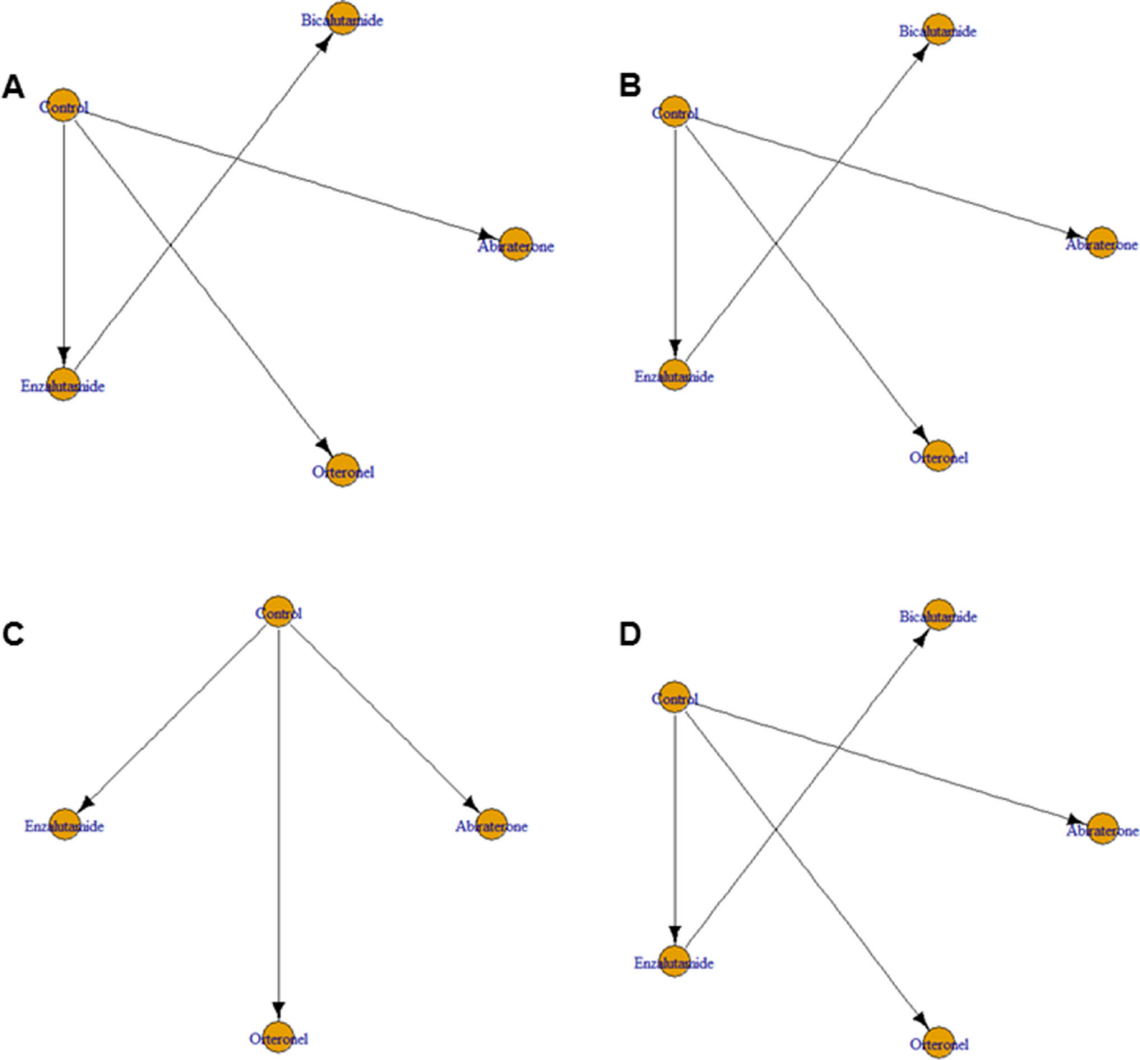
Network geometry of eight randomized controlled trials of novel drugs targeting the androgen receptor signaling pathway for (**A**) progression-free survival, (**B**) time to prostate-specific antigen (PSA) progression, (**C**) time to first skeletal-related events, and (**D**) development of adverse events in patients with metastatic castration-resistant prostate cancer. Arrows indicate studies that were direct comparisons between the agents shown using yellow circles.

**Figure 3:**
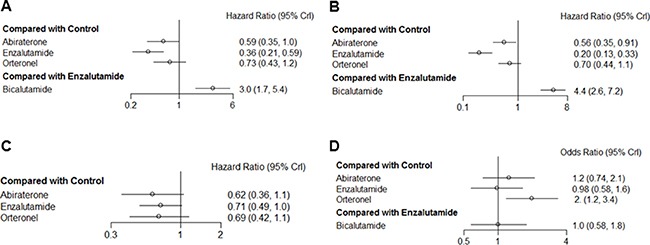
Pooled hazard ratios and 95% credible intervals for the secondary endpoints of our study of patients with castration-resistant prostate cancer (**A**) progression-free survival, (**B**) time to PSA progression, (**C**) time to first skeletal-related events, and (**D**) development of adverse events.

Next, we applied network meta-analysis to compare the secondary endpoints progression-free survival (PFS), time to prostate-specific antigen (PSA) progression, time to first skeletal-related events (SRE), and adverse events (AE) for the AR-targeted drugs. The associated networks are shown in Figure 2. Although enzalutamide markedly improved PFS in comparison with control arms (HR = 0.36, 95% CrI = 0.21–0.59), abiraterone (HR = 0.59, 95% CrI = 0.35–1.0) and orteronel (HR = 0.73, 95% CrI = 0.43–1.2) did not show significant associations with better PFS (Figure 3A). Conversely, bicalutamide treatment increased the risk of progression in comparison with enzalutamide treatment (HR = 3.0, 95% CrI = 1.7–5.4).

Enzalutamide (HR = 0.20, 95% CrI = 0.13–0.33) and abiraterone (HR = 0.56, 95% CrI = 0.35–0.91) were significantly associated with prolonged time to PSA progression, as compared with control groups, whereas orteronel did not have a statistically significant effect on PSA progression time (HR = 0.70, 95% CrI = 0.44–1.11) (Figure 3B). Additionally, the risk of PSA progression was higher for bicalutamide trial arms than for enzalutamide trial arms (HR = 4.4, 95% CrI = 2.6–7.2).

No drugs showed significant effects on the time to first SRE; the 95% CrIs of all three novel AR drugs overlapped with null effect lines, despite nonsignificant associations between the use of these drugs and improvement in this outcome (Figure 3C). Although orteronel was significantly associated with increased AE risk in comparison with controls, abiraterone and enzalutamide groups did not show AE rates that were significantly different from control groups (Figure 3D). Additionally, there was no significant difference between bicalutamide and enzalutamide in terms of AE risk.

For each secondary endpoint (PFS, time to PSA progression, time to first SRE, and AEs), we have provided the detailed results of the rankings for the drugs (Supplementary Tables 2, 3, 4, 5). Additionally, we have presented the results of inconsistency tests, which did not indicate inconsistencies between the direct and indirect evidence for any of the secondary outcomes (Supplementary Figure 2A–2D).

## DISCUSSION

Recently, the landscape of treatments for mCRPC has expanded with the development of several novel AR-targeted drugs, such as abiraterone and enzalutamide [14]. These drugs have shown promising clinical efficacy for mCRPC patients in multicenter Phase III RCTs [8–12]. Particularly, abiraterone and enzalutamide were found to be significantly efficacious for patients with mCRPC in either pre-chemotherapy or post-chemotherapy settings. Further, currently available data suggest that orteronel significantly increases PFS rates, although it has not shown significant associations with OS [8–12, 15]. However, there is a paucity of evidence regarding the comparative outcomes of treatment with different AR-targeted drugs, making it difficult to select the first-line treatment of choice in patients with mCRPC. Notably, no RCT has compared the AR-targeted drugs in a head-to-head fashion.

Roviello *et a*l. [16] published the first pairwise RCT meta-analysis of AR pathway-targeted agents, including eight trials that together enrolled more than 8,500 men with mCRPC. They showed that the new AR pathway-targeted agents decreased the risks of all-cause death and disease progression in mCRPC by 21% and 52%, respectively, in comparison with control groups. Despite the novelty of their meta-analysis, the authors were unable to compare the efficacies of the AR-targeted agents with each other. To provide this information, we performed a network meta-analysis of three orally administrated, novel AR-targeted agents (abiraterone, enzalutamide, and orteronel) for mCRPC patients. To the best of our knowledge, this is the first report to comprehensively compare and rank the efficacies of these drugs by applying network meta-analysis.

Importantly, for the primary endpoint of overall survival, we found that enzalutamide was the most efficacious drug (HR = 0.71), followed by abiraterone (HR = 0.78). Orteronel did not show a significant effect on OS (HR = 0.90). Enzalutamide was also the most efficacious drug for secondary endpoints, particularly PFS (HR = 0.56) and time to PSA progression (HR = 0.20). Additionally, AE risks did not differ between enzalutamide and control arms, suggesting that enzalutamide is safe for clinical use in mCRPC patients. Based on these pieces of evidence together, enzalutamide can be the most efficacious and safe agents for patients with mCRPC and abiraterone can be the second most efficacious drug. Conversely, orteronel had both the least efficacy and was associated with higher AEs. This is the key finding of our study.

We speculate that the three drugs’ different modes of action may have resulted in different survival outcomes. Mechanistically, abiraterone inhibits two specific enzymes (17α-hydroxylase and C17,20-lyase) that are needed for testosterone synthesis from cholesterol precursors. Orteronel blocks enzyme activities of CYP17A1 in the testis, adrenal gland, and prostatic cancer tissues, resulting in significant reductions of circulating testosterone levels in blood [17]. Enzalutamide selectively inhibits AR activities by interfering with different portions of the AR pathway, including nuclear translocation, DNA binding on a promoter region, and interplay with co-activators [18]. Accordingly, as compared with abiraterone and orteronel, enzalutamide may have more selective effects on the AR signaling pathway in prostate cancer cells.

Nonetheless, because our network meta-analysis synthesized evidence from eight studies, differences in study designs, populations, types of control arms, and follow-up durations may have affected the primary and secondary endpoints. For instance, the COU-AA-301 and 302 trials for abiraterone had active control arms that were prescribed prednisone, whereas the PREVAIL and AFFIRM trials for enzalutamide had true control arm that were treated by placebo [16]. Because therapy with prednisone alone has some modest clinical efficacy, differences in final outcomes may have remained between treatment arms.

This study has some limitations. First, we were unable to determine the mechanisms that explained why enzalutamide appeared to be the most efficacious AR-targeted drug. Second, because of the small number of RCTs, we were unable to compare the efficacies of pre-chemotherapy vs. post-chemotherapy administration of AR-targeted drugs. This unavoidable limitation may make it difficult to apply our findings in real-world practice. Third, uncontrolled clinical parameters in the included studies may have distorted the network meta-analysis findings. Despite their prospective nature, there are potential drawbacks to RCTs, including inadequate sample sizes and follow-up periods, heterogeneous disease statuses among enrolled patients, and discrepancies between details of clinical practice among enrolled institutions. Finally, considering that we used random-effects models for the network meta-analyses, studies with relatively small populations, such as the TERRAIN and STRIVE trials, may have had disproportionate influences on the results, as compared with other studies that had larger study populations [19]. Nevertheless, this is the first study to apply network meta-analysis and thereby provide a meaningful comparison of the efficacy and safety of novel AR-targeted drugs.

In summary, our results demonstrated that enzalutamide was the most efficacious of the investigated novel AR-targeted drugs for patients with mCRPC, and was not associated with a significantly elevated risk of side effects. Abiraterone was identified as the second most efficacious drug, while orteronel had both the least efficacy for survival outcomes and was associated with significantly increased AEs. These conclusions were reached by analyzing eight RCTs using network meta-analysis within a Bayesian framework. Although head-to-head RCTs that compare the efficacies of these agents are still needed to acquire more definitive evidence, our work can help to guide the selection of novel AR-targeted drugs for patients with mCRPC.

## MATERIALS AND METHODS

### Search methods

Our meta-analysis was limited to RCTs published before June 2016. In July 2016, Roviellio *et al*. published the first pooled analysis of eight RCTs of novel AR pathway-targeted drugs [16], and we therefore decided to use these eight RCTs for our network meta-analysis [8–12, 15, 20, 21]. Roviellio *et a*l.'s meta-analysis was based on a search of 1,301 RCTs published in PubMed, the Cochrane Library, and the abstracts of American Society of Clinical Oncology meetings on or before January 31, 2016, as reported according to PRISMA guidelines [22].

### Eligibility criteria

This study had the following eligibility criteria, as described according to the PICO strategy: Population: men diagnosed with mCRPC; Intervention: novel drugs targeting the AR signaling pathway (abiraterone acetate, enzalutamide, and orteronel); Control: placebo only, prednisolone only, or bicalutamide only as a traditional anti-androgen; Outcomes: OS as the primary endpoint, and PFS, time to PSA progression, time to first SRE, and AE as secondary endpoints.

### Data extraction and synthesis

Two independent reviewers (C.K. and H.H.K.) extracted data from eligible studies, including the first author, year of article publication, geographic location, study period, numbers of patients in the trial arms, median (or mean) age of patients with mCRPC, names and dosages of anti-androgen drugs, observation periods, and rates and times of the following events: all-cause mortality, disease progression, PSA progression, first SRE, and AEs.

The Cochrane risk-of-bias method was used to assess potential trial biases, based on random sequence generation, randomized allocation sequence concealment, blinding of participants and outcome assessment, incomplete outcome data, selective outcome reporting, and other potential sources of bias [23]. Approval from our institutional review board and written informed consent from enrolled patients were waived because this study was based on data from published articles.

### Statistical analysis

To examine the relative efficacies of the three AR-targeting drugs (abiraterone, enzalutamide, and orteronel), the trial control arms (receiving placebo, prednisolone, or bicalutamide) were used as reference groups for direct and indirect analyses. We defined OS, the primary outcome, as the time from the first randomization date or first administration of AR-targeted drugs until the death from any cause. We also defined PFS, time to PSA progression, time to first SRE, and time to first AE as the times from the randomization or first treatment date until the occurrences of the respective secondary outcomes.

We used HR and CrI from Cox regression analyses to summarize the treatment efficacy of each AR-targeted agent. CrIs are the Bayesian analog to conventional (frequentist) confidence intervals, and can be interpreted similarly. HRs and variances were extracted or estimated for each randomized comparison. When only the variance of the HR was unavailable, it was estimated using the *P*-value of the associated log-rank test. If neither the HR nor the 95% CrI could be obtained directly from the original studies, we estimated these values using the formula log (HR) = (T1+T2)2/[(E1+E2)T1T2]. Here, E1 and E2 are the numbers of events in each treatment arm, and T1 and T2 indicate the numbers of patients randomly assigned to each treatment arm. The log (HR) was finally calculated as it would have been from the log-rank *P*-values [24]. Finally, if *P*-values of log-rank tests were unobtainable, the ratio of median survival times was used to estimate the HR.

Pairwise meta-analysis was performed using the *Der Simonian-Lair*d random-effects model [25]. Statistical heterogeneity was assessed with I^2^ statistics and *P*-values (< 0.05) within and between studies. Values of ^I^2 above 50%, from 25 to 50%, and lower than 25% were regarded as high, moderate, and low heterogeneity, respectively [26]. Next, a random-effects network meta-analysis was conducted within a Bayesian framework to incorporate direct and indirect data into a single comparison, obtaining pooled estimates using Markov chain Monte Carlo methods [27, 28]. Network plots were generated to demonstrate the comparison scheme for each AR-targeted drug. We further calculated ranking probabilities for each treatment's efficacy (i.e., probabilities that the treatment was most, second-most, or third-most efficacious). Finally, we assessed the degree of inconsistency between direct and indirect sources of evidence using a modified back-calculation approach [29]. We did not assess publication bias because at least ten studies are required for funnel plots and Egger's linear regression test.

Pairwise and network meta-analyses were performed using RevMan statistical software (version 5.0, The Cochrane Collaboration, Copenhagen, Denmark), WinBUGS (version 1.4, MRC Biostatistics Unit, Cambridge, UK) and R (version 3.2.2, R Development Core Team, Vienna, Austria, http://www.R-project.org) with the gemtc package. Two-sided *P*-values less than 0.05 were regarded statistically significant.

## SUPPLEMENTARY MATERIALS FIGURES AND TABLES


