# Supplementary material for: Comparing the clinical efficacy of abiraterone acetate, enzalutamide, and orteronel in patients with metastatic castration-resistant prostate cancer by performing a network meta-analysis of eight randomized controlled trials
Source: Oncotarget. 2017 May 10;8(35):59690–7. doi: 10.18632/oncotarget.17741 (PMC5601768; doi:10.18632/oncotarget.17741)
Supplement: Supplementary file 1 [file oncotarget-08-59690-s001.pdf]

Comparing the clinical efficacy of abiraterone acetate, enzalutamide, and orteronel in patients with metastatic castration-resistant prostate cancer by performing a network meta-analysis of eight randomized controlled trials

Supplementary Materials

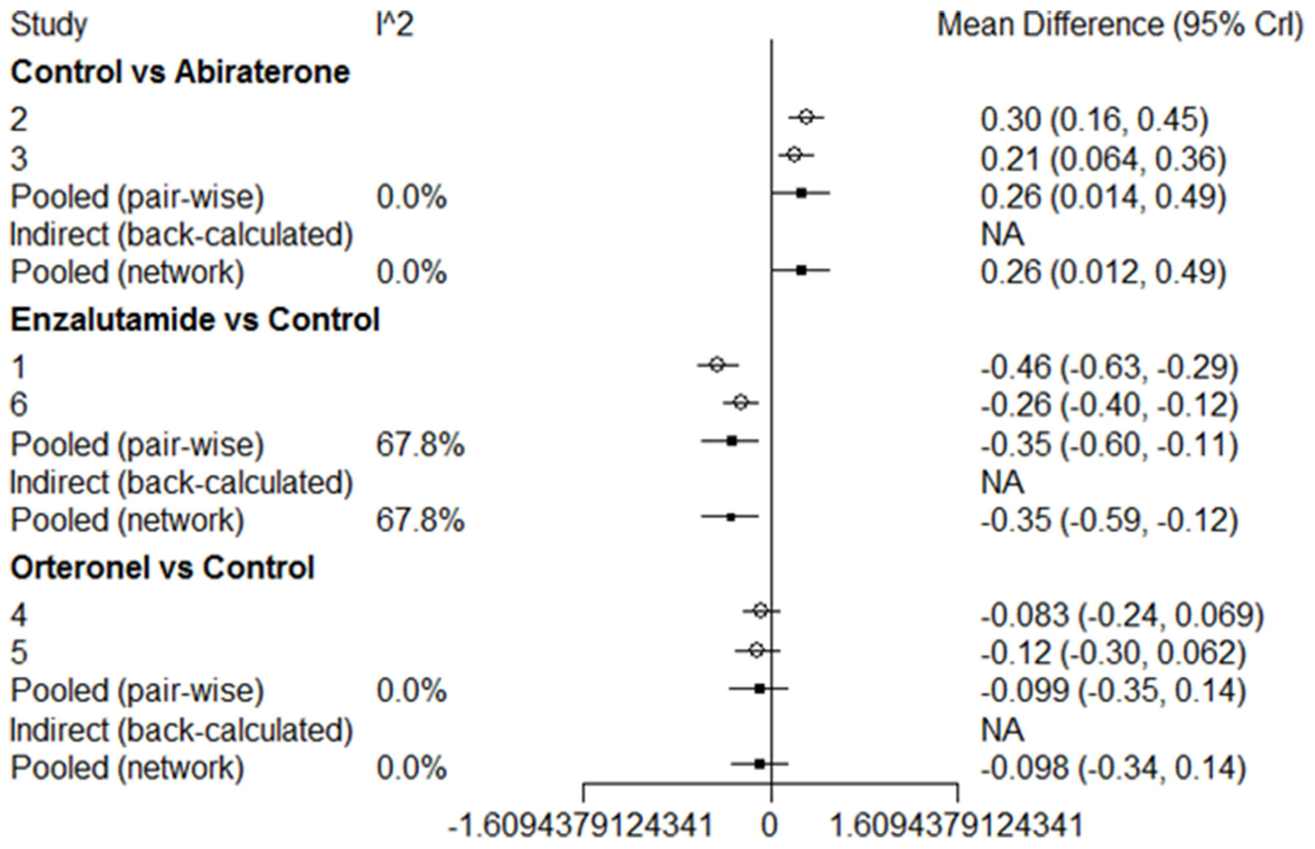

Supplementary Figure 1: Results of inconsistency tests between direct and indirect evidence for overall survival in patients with metastatic castration-resistant prostate cancer.

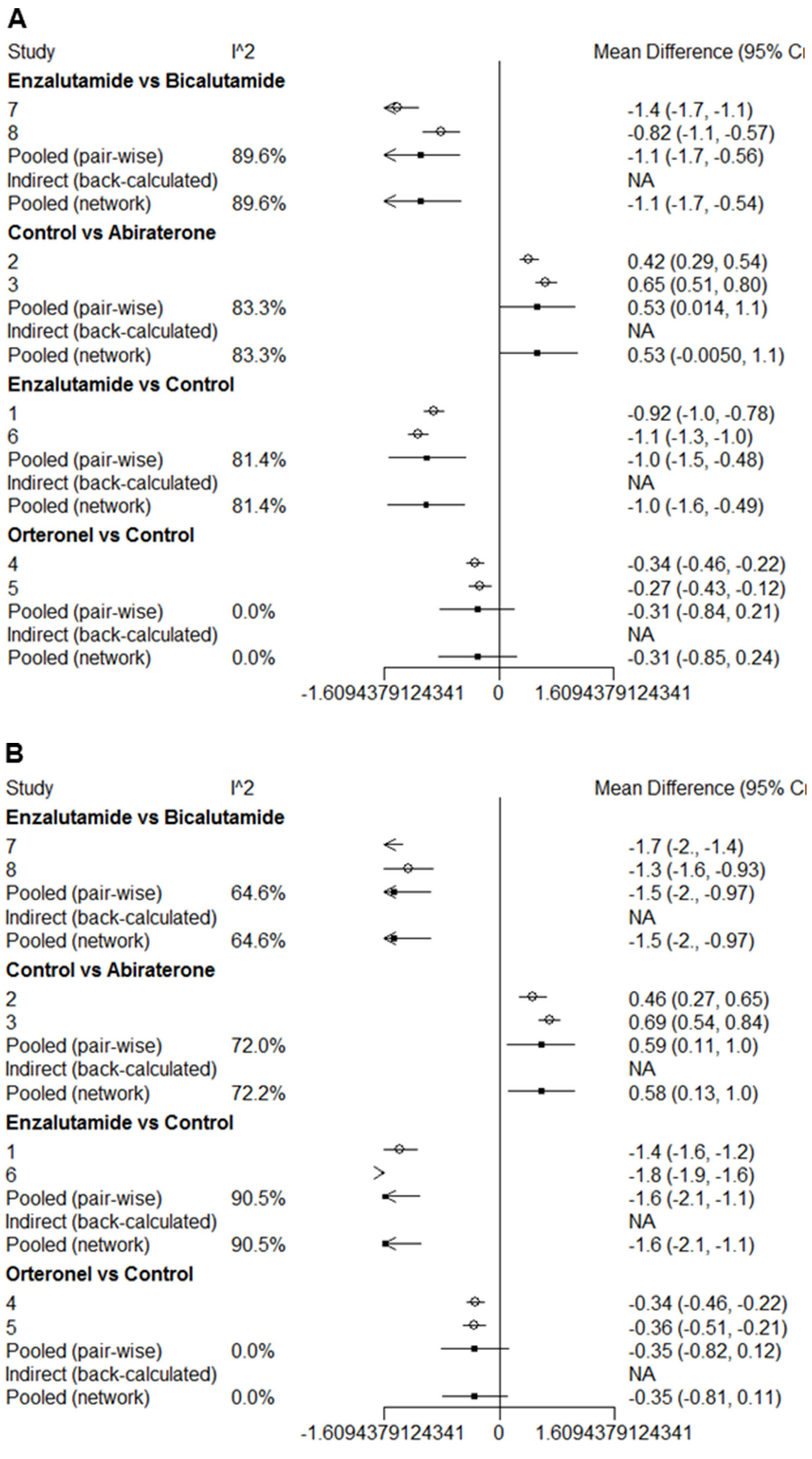
A
Study
I^2
Mean Difference (95% CI
Enzalutamide vs Bicalutamide
7
-1.4 (-1.7, -1.1)
8
-0.82 (-1.1, -0.57)
Pooled (pair-wise) 89.6%
-1.1 (-1.7, -0.56)
Indirect (back-calculated)
NA
Pooled (network) 89.6%
-1.1 (-1.7, -0.54)
Control vs Abiraterone
2
0.42 (0.29, 0.54)
3
0.65 (0.51, 0.80)
Pooled (pair-wise) 83.3%
0.53 (0.014, 1.1)
Indirect (back-calculated)
NA
Pooled (network) 83.3%
0.53 (-0.0050, 1.1)
Enzalutamide vs Control
1
-0.92 (-1.0, -0.78)
6
-1.1 (-1.3, -1.0)
Pooled (pair-wise) 81.4%
-1.0 (-1.5, -0.48)
Indirect (back-calculated)
NA
Pooled (network) 81.4%
-1.0 (-1.6, -0.49)
Orteronel vs Control
4
-0.34 (-0.46, -0.22)
5
-0.27 (-0.43, -0.12)
Pooled (pair-wise) 0.0%
-0.31 (-0.84, 0.21)
Indirect (back-calculated)
NA
Pooled (network) 0.0%
-0.31 (-0.85, 0.24)
-1.6094379124341
0
1.6094379124341
B
Study
I^2
Mean Difference (95% CI
Enzalutamide vs Bicalutamide
7
-1.7 (-2., -1.4)
8
-1.3 (-1.6, -0.93)
Pooled (pair-wise) 64.6%
-1.5 (-2., -0.97)
Indirect (back-calculated)
NA
Pooled (network) 64.6%
-1.5 (-2., -0.97)
Control vs Abiraterone
2
0.46 (0.27, 0.65)
3
0.69 (0.54, 0.84)
Pooled (pair-wise) 72.0%
0.59 (0.11, 1.0)
Indirect (back-calculated)
NA
Pooled (network) 72.2%
0.58 (0.13, 1.0)
Enzalutamide vs Control
1
-1.4 (-1.6, -1.2)
6
-1.8 (-1.9, -1.6)
Pooled (pair-wise) 90.5%
-1.6 (-2.1, -1.1)
Indirect (back-calculated)
NA
Pooled (network) 90.5%
-1.6 (-2.1, -1.1)
Orteronel vs Control
4
-0.34 (-0.46, -0.22)
5
-0.36 (-0.51, -0.21)
Pooled (pair-wise) 0.0%
-0.35 (-0.82, 0.12)
Indirect (back-calculated)
NA
Pooled (network) 0.0%
-0.35 (-0.81, 0.11)
-1.6094379124341
0
1.6094379124341

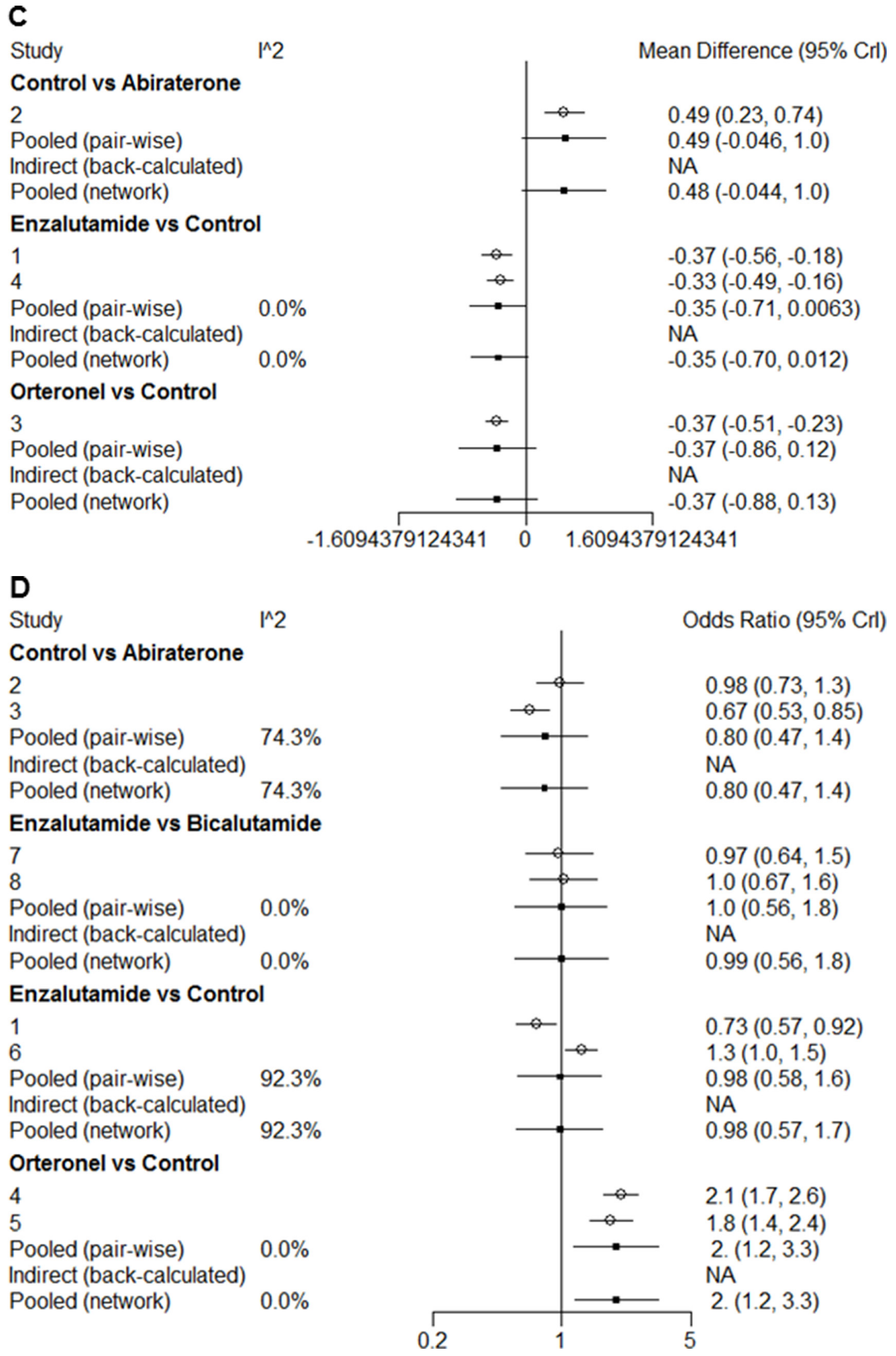

Supplementary Figure 2: Graphs of inconsistency assessments between direct and indirect evidence for (**A**) progression-free survival, (**B**) time to PSA progression, (**C**) time to first skeletal-related events, and (**D**) development of adverse events in patients with metastatic castration-resistant prostate cancer.

Supplementary Table 1: Ranking of overall survival outcomes among the novel drugs targeting androgen receptor signaling pathway in patients with metastatic castration-resistant prostate cancer

*Rank	Abiraterone	Enzalutamide	Orteronel	Control
1	21.00	**75.95**	2.98	0.06
2	**67.28**	20.22	11.68	0.80
3	10.15	3.17	**70.85**	15.82
4	1.56	0.65	14.47	**83.31**

The values correspond with the probabilities that the drug holds the indicated rank (% of 2,000 iterations).
*Lower rank indicates a more efficacious for the noted outcome.

Supplementary Table 2: Ranking of progression-free survival outcomes among the novel drugs targeting androgen receptor signaling pathway in patients with metastatic castration-resistant prostate cancer

*Rank	Abiraterone	Enzalutamide	Orteronel	Bicalutamide	Control
1	4.87	**92.97**	1.96	0.17	0.01
2	**72.26**	5.46	17.73	4.16	0.37
3	18.10	1.32	**64.67**	10.05	5.85
4	3.48	0.22	11.01	25.76	**59.51**
5	1.27	0.01	4.61	**59.85**	34.25

The values correspond with the probabilities that the drug holds the indicated rank (% of 2,000 iterations).
*Lower rank indicates a more efficacious for the noted outcome.

Supplementary Table 3: Ranking of time to prostate-specific antigen progression among the novel drugs targeting androgen receptor signaling pathway in patients with metastatic castration-resistant prostate cancer

*Rank	Abiraterone	Enzalutamide	Orteronel	Bicalutamide	Control
1	0.90	**98.67**	0.36	0.05	0.01
2	**77.62**	1.13	14.25	6.83	0.15
3	16.67	0.13	**63.92**	16.71	2.55
4	3.88	0.05	18.02	46.25	**31.78**
5	0.91	0.01	3.43	**30.15**	65.50

The values correspond with the probabilities that the drug holds the indicated rank (% of 2,000 iterations).
*Lower rank indicates a more efficacious for the noted outcome.

Supplementary Table 4: Ranking of time to first skeletal-related events among the novel drugs targeting androgen receptor signaling pathway in patients with metastatic castration-resistant prostate cancer

*Rank	Abiraterone	Enzalutamide	Orteronel	Control
1	**58.37**	16.66	24.93	0.02
2	22.77	37.81	**38.16**	1.25
3	15.86	**43.31**	31.88	8.93
4	2.98	2.21	**5.01**	89.78

The values correspond with the probabilities that the drug holds the indicated rank (% of 2,000 iterations).
*Lower rank indicates a more efficacious for the noted outcome.

Supplementary Table 5: Ranking of development of adverse events among the novel drugs targeting androgen receptor signaling pathway in patients with metastatic castration-resistant prostate cancer

*Rank	Abiraterone	Bicalutamide	Control	Enzalutamide	Orteronel
1	8.50	**38.12**	27.38	25.60	0.38
2	11.12	18.98	28.86	**40.25**	0.77
3	15.68	20.80	**37.25**	23.83	2.42
4	**57.32**	17.57	6.41	9.26	9.42
5	7.36	4.51	0.08	1.05	**86.98**

The values correspond with the probabilities that the drug holds the indicated rank (% of 2,000 iterations).
*Lower rank indicates a more efficacious for the noted outcome.
